# Types of fish consumption differ across socioeconomic strata and impact differently on plasma fish-based omega-3 fatty acids: a cross-sectional study

**DOI:** 10.1007/s00394-023-03274-x

**Published:** 2023-11-20

**Authors:** Yinjie Zhu, Jochen O. Mierau, Ineke J. Riphagen, M. Rebecca Heiner-Fokkema, Louise H. Dekker, Gerjan J. Navis, Stephan J. L. Bakker

**Affiliations:** 1 Division of Nephrology, Department of Internal Medicine, University Medical Centre Groningen, University of Groningen, Groningen, Hanzeplein 1, 9713 GZ Groningen, The Netherlands; 2 Department of Economics, Econometrics & Finance, Faculty of Economics and Business, University of Groningen, University Complex, 9747 AJ Groningen, The Netherlands; 3 Lifelines Cohort Study and Biobank, Groningen, The Netherlands; 4 Team Strategy & External Relations, University Medical Center Groningen, University of Groningen, Hanzeplein 1, 9713 GZ Groningen, The Netherlands; 5 Certe Medical Diagnostics and Advice, Medical Center Leeuwarden, 8934 AD Leeuwarden, The Netherlands; 6 Laboratory of Metabolic Diseases, University Medical Center Groningen, University of Groningen, Groningen, Hanzeplein 1, 9713 GZ Groningen, The Netherlands; 7 National Institute for Public Health and the Environment (RIVM), 3720 BA Bilthoven, The Netherlands

**Keywords:** Fishes, Omega-3 fatty acids, Ultra-processed food, Socioeconomic status, Food intake

## Abstract

**Purpose:**

We investigated the associations of socioeconomic position (SEP) with total and type of fish intake in a large general population and validated whether types of fish intake were differently associated with plasma EPA and DHA in a subset of the population.

**Methods:**

From the Lifelines Cohort Study, 94,246 participants aged 44 ± 13 years old were included to test the association of two SEP indicators, i.e., education level and household income level, with dietary intakes of total, oily, lean, fried, and other types of fish. In a subset of 575 participants (mean age: 50 ± 13 years), EPA and DHA levels were measured in plasma phospholipids and triglycerides. Dietary fish intake was assessed using Food Frequency Questionnaire. Linear regressions were applied and adjusted for relevant covariates.

**Results:**

Compared to the high education level, lower education levels were negatively associated with total, oily, lean, and other fish intake (*p* < 0.001 for all), and positively associated with fried fish intake (*β* (SE): 0.04 (0.04), *p* < 0.001 for middle education; 0.07 (0.04), *p* < 0.001 for low education), independently of relevant covariates. Similar results were observed for income levels. In the subset population, total and oily fish intakes were positively associated with plasma EPA and DHA (*p* < 0.02 for all). Lean and other fish intakes were positively associated with only DHA (*p* < 0.008 for all), but not EPA, while fried fish was not associated with either EPA or DHA in plasma (*p* > 0.1 for all).

**Conclusion:**

Lower SEP was associated with a lower total intake of fish, and of oily and lean fish, but with higher intake of fried fish. Fried fish was not associated with the fish-based EPA and DHA in plasma. Hence, SEP-related differences in fish consumption are both quantitative and qualitative.

**Supplementary Information:**

The online version contains supplementary material available at 10.1007/s00394-023-03274-x.

## Introduction

Fish intake has been incorporated into several national food-based dietary guidelines because of its associated positive impact on health [1, 2]. The diverse, health-beneficial nutrients in fish, including protein, lipids, vitamins (vitamin D3 and B12), and minerals (iron, iodine, magnesium, potassium, selenium, and zinc), make fish an important food group [3]. Among the various nutrients in fish, poly-unsaturated omega-3 (n-3) fatty acids (FA) hold critical significance, as deficiencies in these FA have been identified as one of the major factors of ill-health globally [4]. More specifically, fish is the major dietary source of n-3 FA eicosapentaenoic acid (EPA) and docosahexaenoic acid (DHA) [4]. According to an intervention review, EPA and DHA have beneficial effects in reducing the risk of coronary heart disease mortality and events and reducing serum triglycerides [5]. Another meta-analysis also found that marine n-3 supplementation lowers the risks for several cardiovascular disease (CVD) outcomes and mortality [6]. Therefore, EPA and DHA, together with other nutrients are in the group of the active nutrients contributing to the favorable health impacts of fish consumption.

While the beneficial effect of EPA and DHA are well-established, observational studies reported inconsistent results regarding the association between total fish consumption and clinical outcomes in the general healthy population. A meta-analysis reported an inverse association between fish consumption and risk of cardiovascular disease (CVD) mortality and all-cause mortality in the general healthy population [7], while another study indicated such association only among patients with prior CVD but not in the general healthy population [8]. These studies proposed future investigations on types of fish intake, as such controversy might be explained by factors such as the type of fish (oily vs. lean), methods for fish preparation (grilled vs. fried), and consumption of other foods associated with lifestyle and socioeconomic position (SEP) [9].

SEP is a crucial determinant of dietary food intake and diet quality [10]. Consumption of foods generally, and of the types and quantity of fish specifically, may differ substantially across socioeconomic strata [11]. While it is well-documented that people with low SEP have lower than average fish intake [12], limited evidence is available regarding the types of fish intake among individuals with different SEP.

In this study, we investigated how fish consumption patterns differ across socioeconomic strata in a general healthy population; importantly, considering that EPA and DHA could indicate the health benefits of fish oil intake and plasma EPA and DHA are valid markers for dietary EPA and DHA intake [13], we also investigated how different fish consumption patterns impact on plasma EPA and DHA in a subset of the population.

## Methods

### Study design and population

The Lifelines Biobank and Cohort Study (Lifelines) is a multidisciplinary prospective population-based cohort study that applies in a unique three-generation design of the health and health-related behaviors of 167,729 persons living in The Netherlands. It employs a broad range of investigative procedures in assessing the biomedical, socio-demographic, behavioral, physical, and psychological factors which contribute to health and disease of the general population. In short, the first group of participants was recruited via local general practitioners. Then participants could indicate whether their family members were interested as well. Additionally, individuals who were interested in the study could register via an online registration system. Individuals with insufficient knowledge of the Dutch language, and with severe psychiatric or physical illness were excluded from the study. Before study entry, a signed informed consent form was obtained from each participant. Adult participants (≥ 18 years) were asked to complete several self-administered questionnaires regarding various aspects, including demographics, socioeconomic status, and lifestyle behaviors. The Lifelines study was conducted according to the principles of the Declaration of Helsinki and approved by the Medical Ethics Committee of the Institutional Review Board of the University Medical Center Groningen, The Netherlands (2007/152). A detailed description of the Lifelines cohort study can be found elsewhere [14, 15].

For the current study, all participants and variables included in this study were from Lifelines baseline assessment that was conducted between 2007 and 2013. In total, 94,246 adult participants from Lifelines baseline assessment with dietary intake data from validated food frequency questionnaire (FFQ) were included to investigate the association of SEP with total and types of fish intake. Because of financial and time constraints, a subset of 864 participants from the Lifelines baseline assessment was randomly selected for plasma EPA and DHA measurements. Cases with missing or invalid data on circulating FA, or dietary intake were excluded, leaving a subset of 575 participants for the investigation of total and types of fish intake with plasma EPA and DHA (Supplementary Fig. S1).

### Dietary assessment

Total and types of fish intake were assessed from a semi-quantitative self-reported FFQ. This FFQ assessed the intake of 110 food items over the last month and was developed and validated by Wageningen University [16, 17]. Frequency categories range from ‘not this month’, ‘1 day per month’, ‘2 to 3 days per month’, ‘1 day per week’, ‘2 to 3 days per week’, ‘4 to 5 days per week’, to ‘6 to 7 days per week’, indicating the portion size and household measurements units. The types of fish intake included in the FFQ and this study were oily fish, lean fish, fried fish, and other fish (Supplementary Description S1). More specifically, fried fish in the FFQ is a type of preparation method included all types of deep-fried fish with whiting dough and is commercially available and accessible in the Dutch food environment and is part of the Dutch food culture. Therefore, the FFQ was specifically developed based on the Dutch context. Since fish is not necessarily consumed every day, we have presented the data on total and types of fish intake as weekly (wk) instead of daily.

Total energy intake was estimated from the FFQ using the 2011 Dutch food composition database (NEVO) [18], as adjustment for total energy intake is usually appropriate in epidemiologic studies to control for potential confounding, reduce extraneous variation, and predict effect of diet-related research [19]. The reliability of FFQ data was assessed using the Schofield equation, which demonstrated the ratio of reported energy intake and basal metabolic rate [20]. Based on the Goldberg-cutoff method, the participants with a ratio below 0.87 or above 2.75 were excluded during the dietary assessment [21] (Supplementary Fig. S1).

### Assessment of SEP

SEP was determined by education and income level, assessed by self-report questionnaires, and coded as categorical variables. The education level was categorized according to the International Standard Classification of Education (ISCED): (1) low (level 0, 1, or 2); (2) middle (level 3 or 4); and (3) high (level 5 or 6) [22]. Income level was based on monthly household net income and was categorized as: (1) low (< 2000 euro/month); (2) middle (2000–3000 euro/month); and (3) high (> 3000 euro/month).

### Assessment of plasma EPA and DHA

Fasting blood and multiple blood samples were collected from all participates including Ethylenediaminetetraacetic acid (EDTA)-plasma samples, and stored at − 80 °C until analyses of FA were carried out. EPA and DHA were measured in plasma phospholipids (PL) and triglycerides (TG) compartments. Analyses of FA were performed at the Department of Laboratory Medicine of the University Medical Center Groningen, the Netherlands, using the methodology described by Hoving et al*.* [23]. In short, total lipids were extracted by the method of Folch et al*.*[24], and subsequently, a shortened version of the method of Kaluzny et al*.* from 1985 was used to isolate plasma PL and TG, using aminopropyl SPE columns for the separation (Isolute, Biotage) [25]. FA were transmethylated with methanolic-HCL into fatty acid methyl esters (FAME). The samples were extracted with hexane and eventually redissolved into 100 µL hexane. 100 µL of internal standard for the quantification of FA in TG (19:0) (19.9 mg/100 mL chloroform–methanol, 2:1 v/v), obtained from Sigma–Aldrich (Zwijndrecht, The Netherlands), were added before isolation of lipid classes (PL and TG). For the quantification of FA in PL, 100 µL of free FA 19:0 (50.0 mg/100 mL methanol), obtained from Larodan (Solna, Sweden), was added after isolation of lipid classes as an internal standard. Aliquots of 2 µL were injected into an Agilent model 6890 gas chromatography equipped with a 200 m × 0.25 mm polar column (CP Select for FAME) and detected with an Agilent 7683 series flame ionization detector. FAME were identified by comparing retention times with those of known standards [Supelco 37 component FAME mix (Sigma-Aldrich)]. The FA were measured according to the method as described by Pranger [13]. EPA and DHA in plasma PL and TG were expressed as a relative percentage of total FA in PL and TG (mol%), respectively.

### Other covariates

Age from Lifelines baseline assessment and sex were included as potential confounding variables in all statistical models [11]. Height and body weight were measured and body mass index (BMI) was calculated as body weight (kg) divided by height squared (m^2^) at baseline. Smoking status and BMI were included as covariates because they are both associated with SEP and dietary intake [26–29]. Smoking status was self-reported and was categorized into never, former, and current smoker.

### Statistical analyses

Participants’ characteristics, including demographics, SEP, dietary intake, and smoking status, were presented for all in both total and subset population, as well as across different education and income levels for the total study population, including participants with missing data on education level and income level. Total and types of fish intake were also presented across tertiles of EPA and DHA in plasma PL and TG. Categorical variables are presented as percentages (%). Continuous variables were shown as mean ± standard deviation (SD) or median (interquartile range [IQR]).

In the total study population, we analyzed the associations of education level and income level with total and types of fish intake using linear regression models separately. First, education level and income level were entered as categorical variables, with high education and high income as reference categories, respectively. Then, *p*-values for trend were also shown by fitting education level and income level as ordinal variables. All models were adjusted for age, sex, BMI, energy intake, and smoking status. Possible interactions between education and income levels were also tested with total and types of fish intake. The modification effect of sex on the association of SEP with total and types of fish intake was also investigated. Missing data on BMI and smoking status were imputed using tenfold multiple imputation.

In the subset population, the associations of total and types of fish intake with EPA and DHA in plasma PL and TG were also investigated using linear regressions, respectively. Total and types of fish intake were categorized into deciles and entered as ordinal variables in the models, and the models were adjusted for age, sex, education level, income level, BMI, smoking status, and energy intake. Missing data on education level, income level, BMI, and smoking status were imputed using ten folded multiple imputation. Sensitivity analyses were conducted regressing education and income levels in the subset population with total and types of fish intake as well as plasma EPA and DHA, adjusting for age, sex, BMI, and smoking status.

All statistical analyses were conducted using Stata, version 13.1 (StataCorp, Texas, USA).

## Results

### SEP and types of fish intake in the total study population

The total study population had a mean age of 44 ± 13 years, a BMI of 25.7 ± 4.1 kg/m^2^, and a median total fish intake was 67.1 (28.2–98.2) g/week (Table 1). Approximately 41.3% was male, 30.5% and 29.0% had high- and low-education level, respectively, and 35.0% and 31.4% had high- and low-income level, respectively (Table 1). With the increase in education or income level, the total, oily, lean, and other fish intake significantly increased, while fried fish intake significantly decreased (*p* < 0.001 for all, Supplementary Table S1).

**Table 1:** Characteristic of total study population and subset population*

	Total study population (*N* = 94,246)	Subset population (*n* = 575)
Total fish, g/week	67.1 (28.2–98.2)	81.0 (36.5–122.7)
Oily fish, g/week	0 (0–29.3)	18.2 (0–39.2)
Lean fish, g/week	8.2 (0–37.4)	21.7 (0–49.2)
Fried fish, g/week	0 (0–36.5)	0 (0–36.3)
Other fish, g/week	0 (0–9.8)	0 (0–15.3)
Age, years	44 ± 13	50 ± 15
Sex, male%	41.3	51.1
BMI, kg/m^2^	25.7 ± 4.1	26.0 ± 4.2
Smoking status, %		
Current	17.2	15.3
Former	33.7	39.8
Never	49.2	44.9
Energy intake, kcal/d	2162.1 ± 583.3	1986.0 ± 632.7
Education, %		
Low	29.0	28.7
Middle	40.5	35.1
High	30.5	36.2
Household Income, %		
Low	31.4	33.9
Middle	33.5	32.5
High	35.0	33.5
Plasma FA, %		
EPA_PL	–	0.9 (0.7–1.1)
EPA_TG	–	0.4 (0.2–0.6)
DHA_PL	–	2.2 (1.7–2.8)
DHA_TG	–	0.8 (0.5–1.2)

*FA* fatty acid, *EPA* eicosapentaenoic acid, *DHA* docosahexaenoic acid, *PL* phospholipids, *TG* triglycerides

^*^Categorical variables are presented as percentages (%). Continuous variables were shown as mean ± standard deviation (SD) or median (interquartile range [IQR])

The associations of education and income with types of fish intake are presented in Table 2. Compared to individuals with a high education level, those with lower education levels were negatively associated with total, oily, lean, and other fish intake (*p* < 0.001 for all), and positively associated with fried fish intake (*β* (SE): 0.04 (0.04), *p* < 0.001 for middle education; 0.07 (0.04), *p* < 0.001 for low education), after adjustments for age, sex, BMI, energy intake, and smoking status (Table 2 and Supplementary Table S2). As for income level, compared to the group with a high income level, those with lower income levels were negatively associated with total, oily, lean, and other fish intake (*p* < 0.001 for all), and positively associated with fried fish intake (*β* (SE): 0.02 (0.04), *p* < 0.001 for middle education; 0.03 (0.04), *p* < 0.001 for high education) (Table 2 and Supplementary Table S2).

**Table 2:** Associations of education or income levels with total and types of fish intake in the total study population*

	Total fish		Oily fish		Fried fish		Lean fish		Other fish	
	*β* (SE)	*p*	*β* (SE)	*p*	*β* (SE)	*p*	*β* (SE)	*p*	*β* (SE)	*p*
Education										
High	0.00 (ref)	–	0.00 (ref)	–	0.00 (ref)	–	0.00 (ref)	–	0.00 (ref)	–
Middle	– 0.10 (0.1)	< 0.001	– 0.12 (0.04)	< 0.001	0.04 (0.04))	< 0.001	– 0.10 (0.04)	< 0.001	– 0.06 (0.02)	< 0.001
Low	– 0.13 (0.1)	< 0.001	– 0.17 (0.05)	< 0.001	0.07 (0.04)	< 0.001	– 0.14 (0.05)	< 0.001	– 0.08 (0.03)	< 0.001
Household Income										
High	0.00 (ref)	–	0.00 (ref)	–	0.00 (ref)	–	0.00 (ref)	–	0.00 (ref)	–
Middle	– 0.06 (0.1)	< 0.001	– 0.08 (0.04)	< 0.001	0.02 (0.04)	< 0.001	– 0.06 (0.05)	< 0.001	– 0.04 (0.03)	< 0.001
Low	– 0.06 (0.1)	< 0.001	– 0.07 (0.05)	< 0.001	0.03 (0.04)	< 0.001	– 0.07 (0.05)	< 0.001	– 0.03 (0.03)	< 0.001

^*^Linear regression models adjusted for age, sex, BMI, energy intake, and smoking status; standard coefficient (β),standard error (SE), and p-value were shown

No multiplicative interaction was found of education and income with total and types of fish intake (data not shown). Sex modified the association of education with oily fish intake (*p*_interaction_ = 0.002), with a higher association magnitude found among females. On the other hand, the associations of income with total and lean fish intake were also modified by sex (*p*_interaction_ = 0.005 and 0.001, respectively), with higher association magnitudes observed among males (Supplementary Table S3).

### Types of fish intake and plasma EPA and DHA in the subset population

The subset population had a mean age of 50 ± 15 years, a BMI of 26.0 ± 4.2 kg/m^2^, and the median weekly total fish intake was 81.0 (36.5–122.7) g (Table 1). Approximately 50.1% was male, 36.2% and 28.7% had high- and low-education level, respectively, and 33.5% and 33.9% had high- and low-income level, respectively (Table 1). Across tertiles of plasma PL and TG EPA, the total, oily, lean and other fish intake increased (*p* < 0.04 for all), while no difference was observed for fried fish intake (*p* = 0.7 and 0.9 for fried fish in PL and TG, respectively) (Supplementary Table S4). The total, oily, lean, and other fish intake also elevated across tertiles of plasma PL and TG DHA (*p* < 0.02 for all). Fried fish intake did not differ across both PL and TG DHA (*p* = 0.2 and 0.6, respectively) (Supplementary Table S4).

The associations of types of fish intake with plasma EPA and DHA levels are presented in Table 3. Total and oily fish intakes were positively associated with EPA and DHA in both plasma PL and TG (*p* < 0.02 for all), with higher *β* found for DHA. Lean and other fish intakes were positively associated with only DHA in both plasma PL and TG (p < 0.008 for all), but not EPA, while fried fish was not associated with either EPA or DHA in plasma (*p* > 0.1 for all). Sensitivity analyses showed that individuals with low SEP, indicated by either education or income, seemed to have lower levels of EPA and DHA (Supplementary Table S5). In addition, corresponding associations were found of SEP with total and types of fish intake in the subset population, consistent with the associations found in the total study population (Supplementary Table S6).

**Table 3:** Associations of total and types of fish intake with eicosapentaenoic acid (EPA) and docosahexaenoic acid (DHA) in plasma phospholipids (PL) and triglycerides (TG) in subset population*

	EPA_PL		EPA_TG		DHA_PL		DHA_TG	
	*β* (SE)	*p*	*β* (SE)	*p*	*β* (SE)	*p*	*β* (SE)	*p*
Total fish	0.17 (0.007)	< 0.001	0.14 (0.006)	0.002	0.28 (0.01)	< 0.001	0.24 (0.01)	< 0.001
Oily fish	0.16 (0.006)	0.001	0.11 (0.005)	0.02	0.34 (0.01)	< 0.001	0.27 (0.008)	< 0.001
Fried fish	0.03 (0.006)	0.6	0.03 (0.005)	0.5	0.07 (0.01)	0.1	0.05 (0.008)	0.3
Lean fish	0.08 (0.006)	0.07	0.08 (0.005)	0.1	0.16 (0.01)	0.001	0.15 (0.008)	0.001
Other fish	0.04 (0.005)	0.3	0.03 (0.005)	0.5	0.20 (0.01)	< 0.001	0.12 (0.008)	0.008

^*^Linear regression models adjusted for age, sex, education, income, BMI, smoking status, and energy intake; standard coefficient (β),standard error (SE), and p-value were shown

## Discussion

In this cross-sectional study conducted in a general healthy adult Dutch population, we found that low SEP, determined either by education or income level, was significantly associated with lower consumption of total, oily, lean, and other fish but higher consumption of fried fish. For a subset of the study population, oily fish was positively associated with both plasma EPA and DHA in PL and TG classes, lean and other fish were positively associated with plasma DHA only, whereas fried fish intake was neither associated with plasma EPA nor DHA in PL and TG classes.

### Comparison with other studies and interpretations

The negative association found between SEP and total fish intake is consistent with previous literature [12, 31]. Yet, little evidence is available regarding SEP and types of fish intake, despite that studies have proposed that intake of fried fish often coincides with lower SEP and types of fish intake is dependent on an individual’s SEP [9, 32]. Our study revealed important variations in types of fish intake across SEP strata. If only total fish intake were considered in a study, it may obscure the fact that individuals with low SEP have a higher intake of fried fish but lower intakes of other types of fish. We also found that education level had a greater impact on the types of fish consumed than income level because of the more pronounced association magnitudes observed. The interactions of sex with education and income levels demonstrated that education or income level impacted fish consumption differently in males and females, i.e., while education yielded a larger impact among females, income was more influential among males.

In the subset population, a null association was found for fried fish intake with both plasma EPA and plasma DHA concentrations, which corresponds with a speculation of a study indicating that fried fish intake might weaken the association between fish-based n-3 FA and coronary calcification in a population aged ≥ 55 y [32]. Another study also reported that only non-fried fish consumption was inversely associated with chronic kidney disease (CKD) incidence [33]. Moreover, it is suggested that commercially prepared fried fish should be avoided because they are low in n-3 FA [34], and the fried fish in our study included all types of deep-fried fish with whiting dough which is typically formulated with food additives to reach the ideal palatability and shelf life in the Dutch food environment. Thus, the fried fish in this study is mostly commercially prepared and could be classified as ultra-processed food (UPF) according to the NOVA food classification system [35]. There is a growing amount of evidence linking UPF with a range of health risks, some of which have proposed that the nutritional composition of the final product could play a role in these detrimental associations [36–39]. Considering that fried fish undergo a deep-frying process at high temperatures multiple times, which could hydrogenate the unsaturated bonds of n-3 FA, we hypothesize that the lipids composition of the original fish product has been modified, as also suggested in a previous study [32]. Plus, recent observational population studies found that only fish rich in n-3 FA was associated with a lower risk of CVD [8, 40], while one study found that other fish was neutrally associated with risk of CVD [8]. Therefore, it is plausible that higher amount of n-3 FA in fish will result in higher levels of n-3 FA in plasma, and subsequently impact health, while fish lower in n-3 FA (e.g., fried fish) could impose little effect on plasma n-3 FA, and thus might contribute less to health outcomes.

### Implications for dietary guidelines, food policies, and public health nutrition advice

According to our results, individuals with low SEP are more likely to consume the type of fish that was not associated with plasma n-3 FA levels, which is also partially confirmed by a systematic review that foods of lower nutritional value tended to be selected by individuals with lower SEP [41]. Nonetheless, this study only demonstrated the unfavored n-3 FA profile of fried fish, and it is vital to acknowledge that fish is a nutritious food group and an excellent source for various other nutrients, such as high-quality protein, vitamin D, vitamin B12, iron, zinc, iodine, and selenium [3]. Additionally, the Netherlands as a whole has low fish intake with only around 23% of the total population meeting the amount recommended by the Dutch dietary guideline and evident socioeconomic gradients being present (Supplementary Table S1), regardless of the types of fish [42]. Thus, overall fish consumption should be encouraged at policy level to the whole Dutch population.

For a tailored and prospective food consumption policy for those with low SEP, towards a healthier choice of fish, a socio-ecological approach is needed to impact the consumption embedded in the food environment [43]. On one hand, further health promotion programs need to be available, highlighting current recommendations for fish consumption and how these targets can be achieved, such as suitable meal plans containing different types of fish to achieve sufficient fish-based n-3 FA within a reasonable budget [44, 45]. On the other hand, food subsidy programs could be applied to promote the consumption of oily and lean fish as they are shown to promote healthy nutrition and reduce SEP inequality in health [48]. Studies have found that subsidizing more nutritious foods tends to be effective in modifying dietary behavior [49]. Moreover, the improved intake of targeted nutrients and foods could potentially reduce the rate of non-communicable diseases in adults if the changes in diet are sustained [50].

In addition, critics of the ultra-processed food concept argue that in modern societies, it is unrealistic to advise people to eliminate ultra-processed fish/foods from their habitual diet and that reformulating the nutrient composition of processed foods is a more effective way to help improve the nutritional value of the foods [52], e.g., EPA and DHA fortified fried fish. Whether or not to advocate the avoidance of ultra-processed fish/food will become more apparent when more evidence about the nutritional composition of UPF is available. This will require a collaborative and joint effort from nutritionists, food scientists, and public health policymakers.

### Strengths and limitations

This study has several strengths. First, using two SEP indicators simultaneously increased the generalization of socioeconomic differences in fish intake. On the other hand, we could establish how these two indicators impact fish intake differently. Second, the total and types of fish intake were derived from an externally validated FFQ developed for Lifelines biobank, which elevated the reliability of the dietary intake data. Third, we have provided insight into whether a type of UPF, i.e., fried fish, could have resulted in different nutritional value in our body. We have validated this via objective assessments of plasma EPA and DHA that serve as biomarkers for fish-based EPA and DHA intake because these two essential n-3 FA are mainly ingested from the diet.

Several limitations should also be noted. First, no causal inferences should be drawn from our findings, given the cross-sectional nature of our study. Second, in the total study population, participants with missing data on education or income were not included in the statistical models, and we have presented the descriptive data of participants with missing education or income in Supplementary Table S7; no substantial differences were observed for people with missing education data, while people with missing income data were likely to have lower total, oily, lean, and other fish intake, but the higher fried fish intake, compared to the total study population. Still, we did not impute the missing income as they are most likely to have a low household income. Therefore, the results will even be more pronounced if we have imputed the missing income data. Third, we were not able to exclude those who took n-3 FA supplementation as this information was not collected at baseline assessment. Fourth, the Lifelines cohort is a single cohort study from a region with a predominantly Caucasian population (more than 99%) in The Netherlands, a country with a well-developed social security system. This may limit its generalizability to populations of other ethnicities and in a different social context.

## Conclusion

SEP-related differences in fish consumption are both quantitative and qualitative, meaning that individuals with lower SEP predominantly consume the type of fish that could have a poor EPA and DHA profile. While the overall fish consumption should be encouraged for the Dutch population, specific nutrition education disseminating avoidance of ultra-processed fried fish and food subsidy programs promoting consumption of oily and lean fish are needed jointly to impact individuals’ food consumption behavior towards types of fish.

### Supplementary Information

Below is the link to the electronic supplementary material.

Supplementary file1 (DOCX 91 KB)

## Data Availability

Data described in the manuscript, code book, and analytic code will not be made available because the authors do not have the authority to share them according to Lifelines data access permissions. But any researchers can apply to use Lifelines data, including the variables used in this investigation. Information about access to Lifelines data is given on their website: (https://www.lifelines.nl/researcher/how-to-apply).
