# Supplementary material for: Types of fish consumption differ across socioeconomic strata and impact differently on plasma fish-based omega-3 fatty acids: a cross-sectional study
Source: Eur J Nutr. 2023 Nov 20;63(2):435–43. doi: 10.1007/s00394-023-03274-x (PMC10899282; doi:10.1007/s00394-023-03274-x)
Supplement: Supplementary file 1 — Supplementary file1 (DOCX 91 KB) [file 394_2023_3274_MOESM1_ESM.docx]

**Title: Types of fish consumption differ across socioeconomic strata and impact differently on plasma fish-based omega-3 fatty acids: A cross-sectional study**

Yinjie Zhu^1*^, Jochen O. Mierau^2,3,4^, Ineke J. Riphagen^5^, M. Rebecca Heiner-Fokkema^6^, Louise H. Dekker^1,7^, Gerjan J. Navis^1^, Stephan J. L. Bakker^1^

^1^ Department of Internal Medicine, Division of Nephrology, University Medical Centre Groningen, University of Groningen, Groningen, Hanzeplein 1, 9713 GZ Groningen, The Netherlands;

^2^ Department of Economics, Econometrics & Finance, Faculty of Economics and Business, University of Groningen, University complex, 9747 AJ Groningen, The Netherlands;

^3^ Lifelines Cohort Study and Biobank, Groningen, The Netherlands;

^4^ Team Strategy &External Relations, University of Groningen, University Medical Center Groningen, Hanzeplein 1, 9713 GZ Groningen, The Netherlands;

^5^ Certe Medical Diagnostics and Advice, Medical Center Leeuwarden, 8934 AD Leeuwarden, the Netherlands;

^6^ Laboratory of Metabolic Diseases, University Medical Center Groningen, University of Groningen, Groningen, Hanzeplein 1, 9713 GZ Groningen, The Netherlands;

^7^ National Institute for Public Health and the Environment (RIVM), 3720 BA Bilthoven, The Netherlands.

*****Correspondence: Yinjie Zhu; Division of Nephrology, Zusterhuis, University Medical Center Groningen, Hanzeplein 1, 9713 GZ Groningen, The Netherlands; y.zhu@umcg.nl; Tel: +31 (0) 626652320

**Supplementary File**


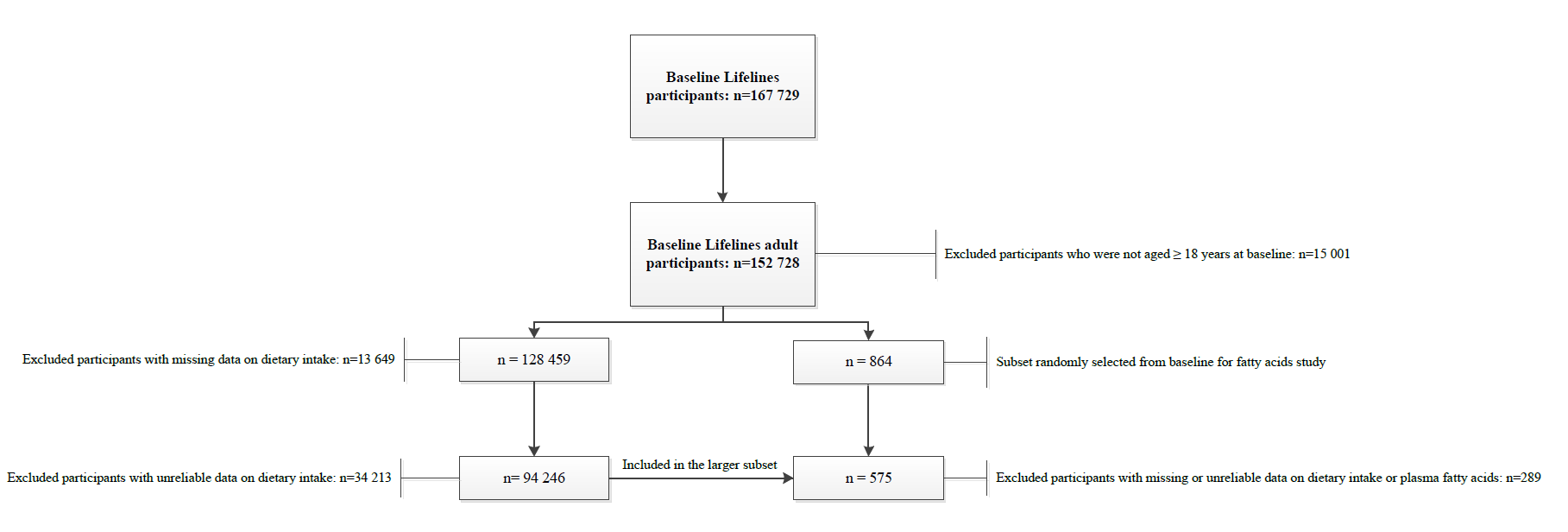


**Supplementary Figure S1. Study flow chart**

| **Supplementary Table S1. Characteristics across education or household income levels of the total study population** | | | | | | | | |
| --- | --- | --- | --- | --- | --- | --- | --- | --- |
|  | Education | | |  | Income | | |  |
|  | Low (n=26782) | Middle (37404) | High (28224) | p | Low (25115) | Middle (26795) | High (27977) | p |
| % Dietary recommendation* | 19.7 | 20.8 | 29.6 |  | 21.6 | 21.6 | 27.0 |  |
| Total fish, g/wk | 60.5 (20.5-91.8) | 61.7 (24.7-92.1) | 77 (32.9-118.7) | <0.001 | 61.1 (18.3-95.7) | 65.1 (27.9-98.1) | 76.2 (32.8-114.6) | <0.001 |
| Oily fish, g/wk | 0 (0-24.3) | 0 (0-29.1) | 18.2 (0-38.7) | <0.001 | 0 (0-29.1) | 0 (0-29.1) | 14.8 (0-36.5) | <0.001 |
| Lean fish, g/wk | 0 (0-32.3) | 0 (0-32.8) | 20.2 (0-43.4) | <0.001 | 0 (0-32.5) | 6.6 (0-32.8) | 20.1 (0-40.8) | <0.001 |
| Fried fish, g/wk | 17.9 (0-36.5) | 9.1 (0-36.5) | 0 (0-30.3) | <0.001 | 0 (0-36.5) | 11.3 (0-36.5) | 0 (0-36.3) | <0.001 |
| Other fish, g/wk | 0 (0-0) | 0 (0-8.2) | 0 (0-15.3) | <0.001 | 0 (0-6.1) | 0 (0-8.2) | 0 (0-12.4) | <0.001 |
| Age, years | 50 ± 13 | 42 ± 12 | 42 ± 12 | <0.001 | 42 ± 15 | 45 ± 12 | 45 ± 10 | <0.001 |
| Sex, male% | 41.2 | 39.8 | 43.3 | <0.001 | 37.0 | 44.5 | 45.9 | <0.001 |
| BMI, kg/m^2^ | 26.6 ± 4.3 | 25.7 ± 4.1 | 24.9 ± 3.6 | <0.001 | 25.6 ± 4.5 | 26.0 ± 4.0 | 25.4 ± 3.6 | <0.001 |
| Smoking status, % |  |  |  |  |  |  |  |  |
| Current | 22.3 | 18.5 | 10.6 | <0.001 | 22.9 | 16.9 | 12.7 | <0.001 |
| Former | 39.9 | 31.6 | 30.3 |  | 28.6 | 36.8 | 35.9 |  |
| Never | 37.8 | 49.9 | 59.1 |  | 48.5 | 46.3 | 51.3 |  |
| Energy intake, kcal/d | 2164.0 ± 615.7 | 2188.7 ± 599.2 | 2126.6 ± 526.1 | <0.001 | 2152.1 ± 612.7 | 2186.7 ± 571.9 | 2156.6 ± 546.2 | <0.001 |

*Categorical variables are presented as percentages (%). Continuous variables were shown as mean ± standard deviation (SD) or median (interquartile range [IQR]). % Dietary recommendation denotes the percentage of people meet the Dutch Dietary Guideline according to the Dutch Healthy Diet Index 2015 (>=105 g/wk).

**Supplementary Table S2. Associations of education or household income level with total and types of fish intake as continuous variables in the total study population.**

|  | Total fish |  | Oily fish |  | Fried fish |  | Lean fish |  | Other fish |  |
| --- | --- | --- | --- | --- | --- | --- | --- | --- | --- | --- |
|  | β (SE) | p | β (SE) | p | β (SE) | p | β (SE) | p | β (SE) | p |
| Education | 0.12 (0.05) | <0.001 | 0.15 (0.02) | <0.001 | -0.06 (0.02) | <0.001 | 0.12 (0.03) | <0.001 | 0.07 (0.01) | <0.001 |
|  | Total fish |  | Oily fish |  | Fried fish |  | Lean fish |  | Other fish |  |
|  | β (SE) | p | β (SE) | p | β (SE) | p | β (SE) | p | β (SE) | p |
| Household Income | 0.05 (0.05) | <0.001 | 0.06 (0.02) | <0.001 | -0.03 (0.02) | <0.001 | 0.06 (0.03) | <0.001 | 0.02 (0.01) | <0.001 |
| *Linear regression models adjusted for age, sex, BMI, energy intake, smoking status | | | | | | | | | | |

**Supplementary Table S3. Modification effect of sex in the associations of socio-economic status (SES) with total and types of fish intake in the total study population.**

| Education | Oily fish |  | Education*Sex | |
| --- | --- | --- | --- | --- |
|  | β (SE) | p | β (SE) | P |
| Male |  |  | 0.04 (0.04) | 0.002 |
| Low | -0.16 (0.07) | <0.001 |  |  |
| Middle | -0.11(0.06) | <0.001 |  |  |
| High | Ref | |  |  |
| Female |  |  |  |  |
| Low | -0.18 (0.06) | <0.001 |  |  |
| Middle | -0.14 (0.05) | <0.001 |  |  |
| High | Ref | |  |  |

| Household Income | Total fish |  | Income*Sex | | Lean fish |  | Income*Sex | |
| --- | --- | --- | --- | --- | --- | --- | --- | --- |
|  | β (SE) | p | β (SE) | P | β (SE) | p | β (SE) | P |
| Male |  |  | -0.04 (0.1) | 0.005 |  |  | -0.05 (0.05) | 0.001 |
| Low | -0.07 (0.2) | <0.001 |  |  | -0.08 (0.08) | <0.001 |  |  |
| Middle | -0.07 (0.1) | <0.001 |  |  | -0.07 (0.07) | <0.001 |  |  |
| High | Ref | |  |  | Ref | |  |  |
| Female |  |  |  |  |  |  |  |  |
| Low | -0.05 (0.1) | <0.001 |  |  | -0.06 (0.07) | <0.001 |  |  |
| Middle | -0.05 (0.1) | <0.001 |  |  | -0.05 (0.07) | <0.001 |  |  |
| High | Ref | |  |  | Ref | |  |  |

| **Supplementary Table S4. Median (IQR) total and types of fish intake across tertiles of eicosapentaenoic acid (EPA) and docosahexaenoic acid (DHA) in phospholipids (PL) and triglycerides (TG), respectively in subset population (n=575)** | | | | | | | | |
| --- | --- | --- | --- | --- | --- | --- | --- | --- |
|  | EPA_PL | | |  | EPA_TG | | |  |
| Range | t1 [0.17-0.75] | t2 [0.76-1.03] | t3 [1.03-4.7] | p | t1 [0.01-0.31] | t2 [0.31-0.52] | t3 [0.52-3.2] | p |
| Total fish, g/wk | 73.5 (29.5-113.8) | 81.0 (36.5-122.7) | 87.5 (58.9-143.4) | <0.001 | 77 (32.6-115.3) | 80 (34.8-119.1) | 86.5 (61.1-154.5) | <0.001 |
| Oily fish, g/wk | 0 (0-36.5) | 12.1 (0-33.0) | 24.3 (0-58.5) | <0.001 | 0 (0-29.5) | 18.2 (0-38.7) | 24.3 (0-58.2) | <0.001 |
| Lean fish, g/wk | 8.2 (0-41.9) | 26.3 (0-54.3) | 25.9 (0-64.2) | 0.02 | 10.1 (0-42.9) | 20.2 (0-43.8) | 27.0 (0-65.6) | 0.005 |
| Fried fish, g/wk | 0 (0-33.1) | 0 (0-36.5) | 0 (0-36.3) | 0.6 | 0 (0-36.5) | 0 (0-36.5) | 0 (0-36.3) | 0.9 |
| Other fish, g/wk | 0 (0-5.6) | 0 (0-13.1) | 0 (0-20.4) | 0.04 | 0 (0-0) | 0 (0-20.4) | 0 (0-19.6) | 0.01 |
|  | DHA_PL | | |  | DHA_TG | | |  |
| Range | t1 [0.64-1.89] | t2 [1.89-2.62] | t3 [2.63-6.58] | p | t1 [0-0.59] | t2 [0.60-1.05] | t3 [1.06-5.36] | p |
| Total fish, g/wk | 60.2 (18.2-90.3) | 81.4 (47.4-123.7) | 91.4 (68.7-155.5) | <0.001 | 71.7 (28.8-97.2) | 81.0 (36.5-118.0) | 91.5 (65.4-181.7) | <0.001 |
| Oily fish, g/wk | 0 (0-24.3) | 18.2 (0-36.5) | 29.3 (5.9-59.1) | <0.001 | 0 (0-29.1) | 17.4 (0-29.5) | 28.2 (0-59.1) | <0.001 |
| Lean fish, g/wk | 0 (0-40.5) | 27.0 (0-54.3) | 26.7 (0-64.6) | <0.001 | 0 (0-40.5) | 25.9 (0-44.7) | 28.7 (0-65.6) | <0.001 |
| Fried fish, g/wk | 0 (0-30.1) | 0 (0-36.5) | 0 (0-36.5) | 0.2 | 0 (0-36.3) | 0 (0-36.3) | 0 (0-36.5) | 0.6 |
| Other fish, g/wk | 0 (0-0) | 0 (0-12.2) | 0 (0-24.6) | <0.001 | 0 (0-0) | 0 (0-15.8) | 0 (0-20.4) | 0.02 |

**Supplementary Table S5. Association of education and income levels with plasma EPA and DHA.**

|  | EPA_PL |  | EPA_TG |  | DHA_PL |  | DHA_TG |  |
| --- | --- | --- | --- | --- | --- | --- | --- | --- |
|  | β (SE) | p | β (SE) | p | β (SE) | p | β (SE) | p |
| Education |  |  |  |  |  |  |  |  |
| Low | -0.3 (0.05) | 0.5 | 0.04 (0.04) | 0.4 | -0.06 (0.09) | 0.2 | 0.02 (0.07) | 0.6 |
| Middle | -0.02 (0.04) | 0.7 | -0.07 (0.04) | 0.1 | -0.07 (0.08) | 0.1 | -0.05 (0.06) | 0.2 |
| High | Ref |  | Ref |  | Ref |  | Ref |  |
| Income |  |  |  |  |  |  |  |  |
| Low | -0.08 (0.05) | 0.1 | -0.07 (0.04) | 0.2 | 0.01 (0.09) | 0.9 | -0.03 (0.07) | 0.6 |
| Middle | -0.03 (0.05) | 0.5 | 0.01 (0.04) | 0.09 | -0.07 (0.09) | 0.1 | -0.04 (0.07) | 0.4 |
| High | Ref | | Ref | | Ref | | Ref | |

*Linear regression models adjusted for age, sex, BMI, and smoking status.

**Supplementary Table S6. Associations of education and income levels with total and types of fish intake in subset cohort.**

|  | Total fish |  | Oily fish |  | Fried fish |  | Lean fish |  | Other fish |  |
| --- | --- | --- | --- | --- | --- | --- | --- | --- | --- | --- |
| Education | β (SE) | p | β (SE) | p | β (SE) | p | β (SE) | p | β (SE) | p |
| Low | -0.10 (0.13) | 0.06 | -0.13 (0.14) | 0.02 | 0.14 (0.15) | 0.006 | -0.14 (0.14) | 0.009 | -0.12 (0.15) | 0.02 |
| Middle | -0.13 (0.12) | 0.01 | -0.09 (0.13) | 0.08 | 0.03 (0.l3) | 0.6 | -0.08 (0.13) | 0.1 | -0.11 (0.14) | 0.03 |
| High | ref | | | | | | | | | |
|  |  |  |  |  |  |  |  |  |  |  |
| Income | β (SE) | p | β (SE) | p | β (SE) | p | β (SE) | p | β (SE) | p |
| Low | -0.15 (0.13) | 0.004 | -0.11 (0.14) | 0.04 | 0.08 (0.15) | 0.1 | -0.21 (0.14) | <0.001 | -0.13 (0.15) | 0.02 |
| Middle | -0.19 (0.13) | <0.001 | -0.06 (0.14) | 0.3 | 0.04 (0.15) | 0.5 | -0.21 (0.14) | <0.001 | -0.14 (0.15) | 0.01 |
| High | ref | | | | | | | | | |

*Linear regression models adjusted for age, sex, BMI, energy intake, and smoking status.

**Supplementary Table S7. Characteristics of participants with missing data on Education or Household Income of the total study population.**

|  | Missing Education: n=1836 | Missing Household Income: n=14359 |
| --- | --- | --- |
| Total fish, g/wk | 66.5 (29.5-98.2) | 61.1 (20.8-97.2) |
| Oily fish, g/wk | 5.9 (0-29.1) | 0 (0-27.2) |
| Lean fish, g/wk | 2.3 (0-35.6) | 0 (0-32.5) |
| Fried fish, g/wk | 12.2 (0-36.5) | 11.2 (0-36.5) |
| Other fish, g/wk | 0 (0-10.1) | 0 (0-6.1) |
| Age, years | 48 ± 14 | 46 ± 12 |
| Sex, male% | 56.6 | 66.0 |
| BMI, kg/m^2^ | 26.0 ± 4.2 | 26.0 ± 4.2 |
| Smoking status, % |  |  |
| Current | 16.4 | 16.4 |
| Former | 35.7 | 32.2 |
| Never | 47.8 | 51.4 |
| Energy intake, kcal/d | 2137.9 ± 577.4 | 2144.4 ± 619.2 |

*Categorical variables are presented as percentages (%). Continuous variables were shown as mean ± standard deviation (SD) or median (interquartile range [IQR]).

**Supplementary Description S1. Original food frequency questionnaires (in English and Dutch)**

-Oily fish:

English: oily fish (such as salmon, tuna, mackerel, eel, white herring) / which types of fish did you eat in the past month?

Dutch: vette vis (zoals zalm, tonijn, makreel, paling, panharing)/ welke soorten vis nam u dan?

-Lean fish:

English: lean (white) fish (such as cod, plaice, haddock, pollack, sole)/ what type of fish did you eat?

Dutch: magere (witte) vis (zoals kabeljauw, schol, schelvis, koolvis, tong)/ welke soorten vis nam u dan?

-Fried fish:

English: deep-fried whiting in dough / what type of fish did you eat?

Dutch: lekkerbekje of kibbeling/ welke soorten vis nam u dan?

-Other fish:

English: other types of fish / which types of fish did you eat in the past month?

Dutch: overige soorten vis/ welke soorten vis nam u dan?
